# Chemical Modification of Pullulan Exopolysaccharide by Grafting Poly(3-hydroxybutyrate-*co*-3-hydroxyvalerate) (PHBHV) via Click Chemistry

**DOI:** 10.3390/polym12112527

**Published:** 2020-10-29

**Authors:** Layde T. de Carvalho, Maria Luiza da S. Paula, Rodolfo M. de Moraes, Gizelda M. Alves, Talita M. Lacerda, Julio C. dos Santos, Amilton M. dos Santos, Simone de F. Medeiros

**Affiliations:** 1 Department of Chemical Engineering, Engineering School of Lorena, University of São Paulo, EEL-USP, Lorena 12602-810, SP, Brazil; laydetcarvalho@gmail.com (L.T.d.C.); maria.luiza.paula@usp.br (M.L.d.S.P.); rodolfommoraes@hotmail.com (R.M.d.M.); gizelda@dequi.eel.usp.br (G.M.A.); amsantos@usp.br (A.M.d.S.); 2 Department of Biotechnology, Engineering School of Lorena, University of São Paulo, Lorena 12602-810, SP, Brazil; talitalacerda@usp.br (T.M.L.); jsant2000@usp.br (J.C.d.S.)

**Keywords:** pullulan, PHBHV, graft copolymers, polysaccharide, chemical modification

## Abstract

Biodegradable and biocompatible copolymers have been often studied for the development of biomaterials for drug delivery systems. In this context, this work reports the synthesis and characterization of a novel pullulan-*g*-poly(3-hydroxybutyrate-*co*-3-hydroxyvalerate) (Pull-*g-*PHBHV) graft copolymer using click chemistry. Well-defined and functional pullulan backbones containing azide groups (PullN_3_) previously prepared by our group were successfully used for this purpose and propargyl-terminated poly(3-hydroxybutyrate-*co*-3-hydroxyvalerate) was prepared via transesterification using propargyl alcohol as a chain transfer agent. By an alkyne-azide cycloaddition reaction catalyzed by copper (Cu (I)) (CuAAC), the graft copolymer Pull-*g*-PHBHV was obtained. The chemical structures of the polymers were accessed by ^1^H NMR and ^13^C NMR FTIR. Disappearance of the bands referring to the main bonds evidenced success in the grafting reaction. Besides that, DRX, DSC and TGA were used in order to access the changes in crystallinity and thermal behavior of the material. The remaining crystallinity of the Pull-*g*-PHBHV structure evidences the presence of PHBHV. Pull*-g-*PHBHV presented lower degradation maximum temperature values than the starting materials, indicating its minor thermal stability. Finally, the synthesized material is an innovative biopolymer, which has never been reported in the previous literature. It is a bio-derived and biodegradable polymer, chemically modified, resulting in interesting properties which can be useful for their further applications as biomedical systems for controlled delivery, for example.

## 1. Introduction

Polymers from renewable sources are interesting materials due to their unique properties and wide applications. In the biomedical area, surgical sutures, tissue engineering and therapeutic agent delivery devices are typical uses for this technology [1,2]. However, due to the limitation in finding all the required properties in a single homopolymer or polymeric family, it is usual to find the combination of different macromolecules in order to obtain a suitable material for a specific request [3,4,5]. Besides that, polymer modification stands out for creating materials capable of forming important structures such as nanogels, hydrogels and micelles, impacting on the requirements of nanocarrier systems [6]. Amongst the bio-based polymers, polysaccharides and poly(hydroxyalkanoates) are biodegradable and biocompatible materials which are becoming progressively more important despite their relatively early stage of development and commercialization [7].

Pullulan is a polysaccharide produced by a yeast-like fungus *Aureobasidium pullulans,* composed of maltotriose units connected by α-(1-6) linkages [8]. In the human body, pullulan can degrade without activating the immunologic system [9,10]. Therefore, its biocompatibility, non-toxicity, biodegradable, hydrophilic and nonionic characteristics contribute to various researches for biomedical applications such as drug and gene delivery, tissue engineering and drug-controlled activation systems. [11,12]. However, this polysaccharide is not self-assembled in aqueous solution due to its high solubility in water [13]. Thus, aiming its application to controlled drug delivery systems, a partial modification of pullulan with hydrophobic materials is necessary, which can result in amphiphilic properties, allowing to obtain self-assembled nanoparticles for delivering drugs [14,15].

Chemical modification helps in combining specific characteristics of different polymers, resulting in a unique material with interesting properties [3]. A modified polysaccharide is often required in order to combine the advantages of a natural polymer with other substances, obtaining a single material with interesting solubility, biodegradability, thermal and mechanical stability [16,17,18]. Using homogeneous chemical modification, different groups of functions can react with the hydroxyl groups present in the backbone of polysaccharides by esterification, etherification, nucleophilic displacement and graft reactions, resulting in a specific structure such as an amphiphilic material [19,20]. Amphiphilic polysaccharide derivatives are advantageous materials that could self-assemble into nanoparticles in an aqueous solution, avoiding the use of organic solvents and providing the encapsulation and release control of drugs [21,22].

Poly(3-hydroxybutyrate*-co*-3-hydroxyvalerate) (PHBHV) is a biodegradable, biocompatible, non-toxic, hydrophobic material produced from renewable sources by the bacterium *Ralstonia eutropha* [23,24]. This biopolymer belongs to the poly(hydroxyalkanoates) (PHAs) family, that corresponds to aliphatic poly-esters produced by a variety of microorganisms as intracellular carbon and energy reserve materials [25]. The advantage of PHBHV over biopolymers is the encapsulation time for anticancer drugs as an effective barrier that protects the drug from premature degradation [26]. Besides that, it has been intensively reported as an important biomaterial for biomedical devices in tissue engineering developments and controlled release drug delivery systems, mainly because of two reasons: its great potential for obtaining micro and nanocarriers for drug delivery and its low production costs [27,28].

In the present study, we report the preparation of chemically modified pullulan by grafting poly(3-hydroxybutyrate-*co*-3-hydroxyvalerate) onto its backbone. The developed grafted copolymers demonstrated a great potential for drug encapsulation systems. The copolymer was prepared via a 1,3-dipolar alkyne-azide cycloaddition reaction, also known as a click chemistry reaction, catalyzed by copper (Cu(I)). Propargyl-terminated-PHBHV was synthesized by transesterification using propargyl alcohol as a chain transfer agent. Afterwards, it was grafted onto the azide-functionalized pullulan PullN_3_ previously synthesized by our group [29]. The final grafted copolymer demonstrated to be a promising biodegradable and biocompatible material for drug delivery systems, which combine pullulan and PHBHV characteristics, enhancing their physicochemical properties. Furthermore, the copolymer Pull-*g*-PHBHV is an innovative biopolymer synthesized by the versatile method, click chemistry, a process that has never been reported in the previous literature.

## 2. Materials and Methods

### 2.1. Materials

**Figure 1:**
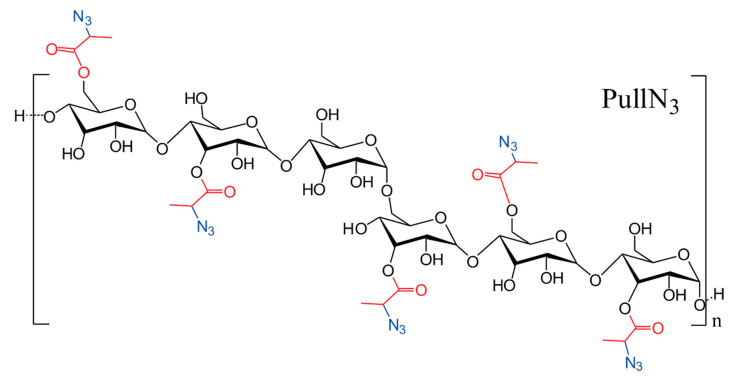
Chemical structure of PullN_3_ used in this work.

Figure 1 presents the chemical structure of PullN_3_ used in this work, with a degree of substitution of DS = 0.08, previously prepared and characterized as described in our last publication [29]. Poly(3-hydroxybutyrate-*co*-3-hydroxyvalerate) (PHBHV, 8.6% mol HV, produced by controlled fermentation process using microorganisms) with an average numerical molar mass (*Mn*) of 159,710 Da and dispersion (*Ð*) equal to 2.86, was purchased by Sigma-Aldrich (St. Louis, MO, USA). Tin dibutyl dilaurate (DBTB, 95%, Sigma-Aldrich, São Paulo, Brazil), copper bromide (CuBr, 98%, Sigma-Aldrich), *N*, *N*, *N*′, *N*’‘, *N*’’-pentamethyldiethylentriamine (PMDTA, 99%, Sigma-Aldrich), propargyl alcohol (99%, Sigma -Aldrich), deuterated dimethyl sulfoxide (DMSO-d6, 99.9%, Sigma-Aldrich) and deuterated chloroform (CDCl_3_, 99.8%, Sigma-Aldrich) were used as received. Toluene (P.A., Labsynth, São Paulo Brazil) was distilled at atmospheric pressure in the presence of calcium hydride (CaH_2_). N, N-dimethylformamide (DMF, 99.8%, Labsynth) was distilled under reduced pressure in the presence of calcium hydride (CaH_2_), and the water used in the entire process was distilled and deionized. Other solvents used in the polymer purification steps, such as, ethyl alcohol (P.A., Labsynth), methyl alcohol (P.A., Labsynth) and ethyl ether (P.A., Labsynth), were used without any prior purification.

### 2.2. Synthesis of Propargyl-Terminated Poly(hydroxybutyrate-co-hydroxyvalerate)

**Scheme 1:**
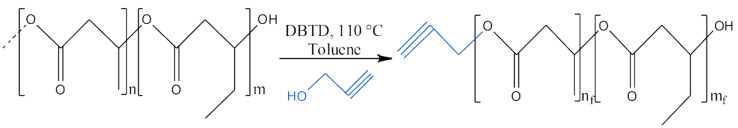
Synthesis of propargyl-terminated PHBHV via transesterification.

For the propargyl-terminated PHBHV, the methodology used was adapted from Lemechko et al., 2012 [30] (Scheme 1). Amounts of 20 g PHBHV (0.125 mmol) and 21.2 mL propargyl alcohol (0.367 mol) were mixed with 270 mL toluene in a round-bottom flask. The mixture was submitted under nitrogen atmosphere and stirred until its complete solubilization. It was then submersed in a glycerin bath at 110 °C, and 1 g solution of dibutyltin dilaurate (1.5 mmol) was added in 30 mL toluene, starting the transesterification reaction. After 12 h, the system was cooled and precipitated in cold n-hexane and kept for 24 h at 0 °C. A white precipitate was obtained by filtration and vacuum-drying.

### 2.3. Synthesis of Pull-g-PHBHV Grafted Copolymer

**Scheme 2:**
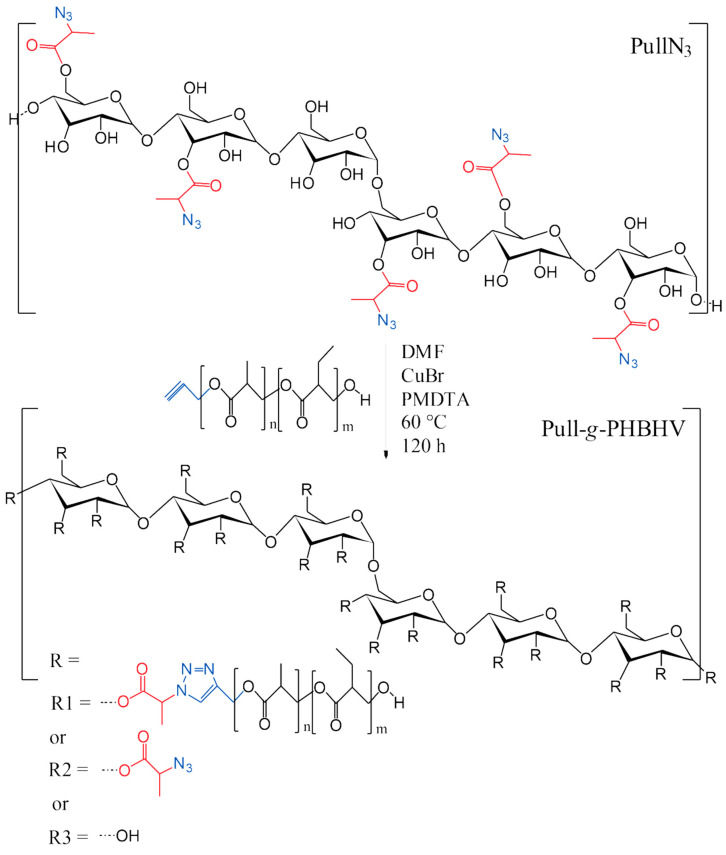
Cycloaddition reaction between azide-functionalized pullulan (PullN_3_) and propargyl-terminated poly(hydroxybutyrate-co-hydroxyvalerate).

The grafting of PHBHV-alkyne onto the PullN_3_ backbone was obtained via a 1,3-dipolar cycloaddition reaction between azide and alkyne, using a Cu (I) catalyst, as shown in Scheme 2.

Initially, 0.0803 g PullN_3_ (0.039 mmol of azide units) was added in a round-bottom flask with 0.1593 g PHBHV-alkyne (0.039 mmol) and 8 mL DMF. After complete dissolution, under nitrogen atmosphere and vigorous stirring, 2.5 mL PMDTA solution (171.4 mg·mL^−1^ PMDTA in DMF) and 2.5 mL CuBr solution (141.88 mg·mL^−1^ CuBr in DMF) were added to the system. The flask was sealed and the reaction mixture was stirred for 120 h at 60 °C. The reaction mixture was precipitated in ethanol, just after finishing the reaction time. The precipitate was filtrated and solubilized again in DMF, which was dialyzed against distilled water for 3 days and lyophilized.

### 2.4. Characterizations

#### 2.4.1. Proton Nuclear Magnetic Resonance (^1^H NMR)

The chemical structures of the modified PHBHV were assessed by proton nuclear magnetic resonance (^1^H NMR) on a Mercury VX 300 spectrometer (Varian, Palo Alto, CA, USA) operating at 300 MHz. The solvents used were DMSO-d_6_ and CDCl_3_, according to the specific solubility of each polymer.

#### 2.4.2. Carbon-13 Nuclear Magnetic Resonance (^13^C NMR)

^13^C solid-state NMR analysis was performed on a Varian Inova spectrometer (Varian, Palo Alto, CA, USA), with frequencies of ^13^C at 100.5 MHz, with the objective of accessing the chemical structure of the grafted copolymer Pull-*g-*PHBHV, as well as comparing with PullN3 and PHBHV-alkyne. The measurements were taken using a 7 mm Jackobsen probe with double resonance and a magic rotation angle. All experiments were performed with a rotation of 10 kHz around the magic angle, with contact times for cross-polarization of 1 ms and time of 5 s between acquisitions.

#### 2.4.3. Gel Permeation Chromatography (GPC)

The numbers of average molecular weight (*Mn*) and dispersity (*Đ*) of the unmodified PHBHV and the propargyl-terminated PHBHV were determined by gel permeation chromatography (GPC), using a Breeze (Waters GPC, USA) chromatograph equipped with a 2414 refractive index (RI) detector. Chloroform with 0.3% v/v of triethylamine was used as the eluent. The sample was prepared with a concentration of 10 mg·mL^−1^, filtered on a modified polyvinylidene difluoride (PVDF) membrane filter (0.45 μm) and subsequently injected into the equipment. Solvent flow rate was 1 mL·min^−1^, and the internal temperature of the columns was 30 °C. Calibration curves were obtained using monodispersed polystyrene standards.

#### 2.4.4. FTIR

The Fourier transform infrared technique was used to identify the polymer organic functions by a qualitative assessment from a Spectrum 100 FTIR (PerkinElmer, Waltham, MA, USA) equipped with an attenuated total reflection accessory (ATR). Diamond crystal was used as the internal reflection element, and resolution of the analysis was 4 cm^−1^.

#### 2.4.5. Thermal Analysis

Thermal properties of the copolymers were evaluated using thermogravimetric analysis (TGA) and differential scanning calorimetry (DSC). The DSC measurements were performed with TA Instruments’ (USA) Q20 equipment. The samples were heated in a rate of 10 °C·min^−1^, from −80 to 200 °C under nitrogen atmosphere. Thermograms were obtained by the second heating cycle in order to eliminate the influence of water in the samples. TGA was performed on the STA 449 F3 Jupiter Netzsch equipment. About 12 to 15 mg of the samples was weighed and submitted to heating from 30 to 700 °C, with heating rates of 10 °C·min^−1^, under nitrogen atmosphere.

#### 2.4.6. X-ray Diffraction

X-ray diffraction patterns were obtained by a PAN-analytical Empyrean ACMS 101 (Malvern, UK) diffractometer at room temperature. The X-ray tube consisted of a target material made of copper (Cu), which emits Kα radiation. Analyses were performed using a 40 kV accelerating potential and current of 40 mA. Experiments were conducted with a scan range from 10 to 60° (2θ), a step size of 0.02° (2θ) and a counting time of 50 s per step.

## 3. Results and Discussion

### 3.1. Propargyl-Terminated Poly(3-hydroxybutyrate-co-3-hydroxyvalerate)

**Figure 2:**
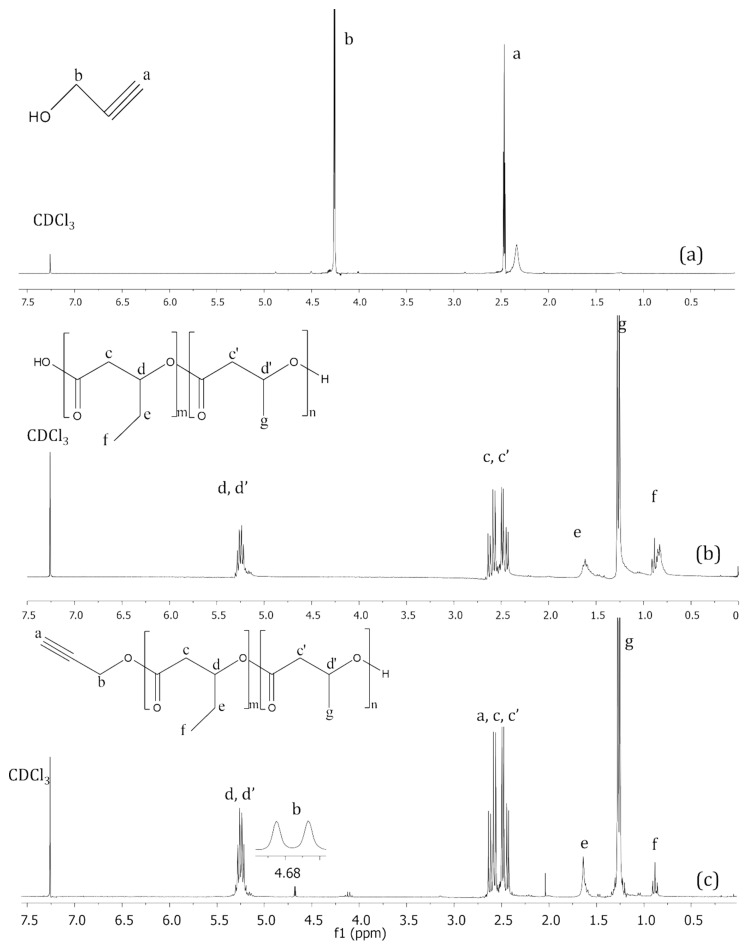
300 MHz ^1^H NMR spectra of propargyl alcohol (**a**), unmodified PHBHV (**b**) and PHBHV-alkyne after transesterification with propargyl alcohol (**c**) in CDCl_3_.

The ^1^H NMR spectra of propargyl alcohol, unmodified PHBHV and PHBHV-alkyne are shown in Figure 2a–c, respectively, while molar masses and dispersity (*Đ*) of the unmodified PHBHV and PHBHV-alkyne are listed in Table 1. The ^1^H NMR spectrum shown in Figure 2a is characteristic of a propargyl group in accordance with the literature [31,32], exhibiting resonances (ppm) at chemical shifts 4.25 (2H, HC≡C−CH_2__−_, represent by “a”) and 2.47 (1H, HC≡C−CH_2_−, represented by “b”). In Figure 2b, the ^1^H NMR spectrum shows resonances from 5.20 to 5.28 (1H, -CH- of HB and HV unities, represented by “d” and “d′ ”, respectively), from 2.42 to 2.66 (2H, -CH_2_- of HB and HV unities, represented by “c” and “c′ ”, respectively), 1.64 (2H, -CHCH_2_CH_3_ of HV, represented by “e”), 1.26 (3H, -CHCH_3_ of HB, represented by “g”) and 0.88 (3H, -CHCH_2_CH_3_ of HV, represented by “f”), all referring to the protons of the main polymer chain before its transesterification [33]. When compared to the spectrum presented in Figure 2c, the characteristic resonances of PHBHV are observed, but the presence of the proton from the propargyl alcohol molecule bonded at the end of the PHBHV chain at chemical shift 4.68 (2H, HC≡C-CH_2_-, represented by “b”), which is absent in the spectrum shown in Figure 2b, corroborates the PHBHV functionalization with alkyne groups.

Data shown in Table 1 provide evidence about the success of the PHBHV transesterification reaction in the presence of propargyl alcohol. The molar mass was determined by two different methods, GPC and ^1^H NMR, resulting, respectively, in 4049 and 2843 Da. The main reason for the difference between the values is due to the polystyrene standard used in the GPC analysis, which does not have the same hydrodynamic volume in THF. In order to calculate the molar mass of the PHBHV-alkyne, an integration value of 1.00 was attributed to the ^1^H NMR signal at 4.68 ppm (Figure 2c) from the propargyl unit. After, the signals at 0.88 and 1.26 ppm (represented by “f” and “g” in Figure 2c) were integrated, resulting in 4.61 and 43.28, respectively. This integration values were proportionally multiplied to the molecular weight of the respective monomer, summing up after it. Then, the molecular weight of the propargyl unit was added to the previous calculation, referring to the propargyl group present at the end of the polymer chain. Analyzing the GPC results, it can be notice that the *Mn* value of PHBHV-alkyne reduced significantly from 114,873 to 4049 Da, when compared with the data from unmodified PHBHV. In addition, the decreased dispersity (*Đ)* from 2.86 to 1.54 demonstrated the homogeneity of the copolymer chains after transesterification. Thus, it is expected that these interesting results represent the potential application of PHBVH-alkyne for future biomedical applications.

**Figure 3:**
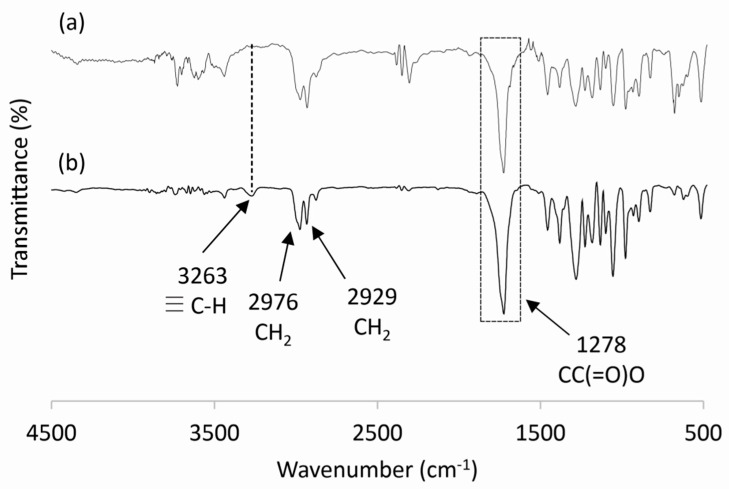
FTIR spectra of unmodified PHBHV (**a**) and PHBHV-alkyne after transesterification with propargyl alcohol (**b**).

The FTIR spectra of unmodified PHBHV and alkyne-functionalized PHBHV (PHBHV-alkyne) have both presented stretching at 1722 cm^−1^ related to carbonyl groups of the PHBHV. The bands at 2976 and 2929 cm^−1^, present in both spectra, are characteristic of the asymmetric and symmetric stretching of the C-H bonds, respectively. The asymmetric stretching of the CC(=O)O ester group of PHBHV is characterized by the band at 1278 cm^−1^. The band at 3263 cm^−1^, present only in the spectrum shown in Figure 3b, is characteristic of the axial stretching of the H-C≡ bond and therefore characterizes the PHBHV functionalization with alkyne groups.

### 3.2. Grafting of PHBHV-Alkyne onto PullN_3_ Chains via Click Chemistry

**Figure 4:**
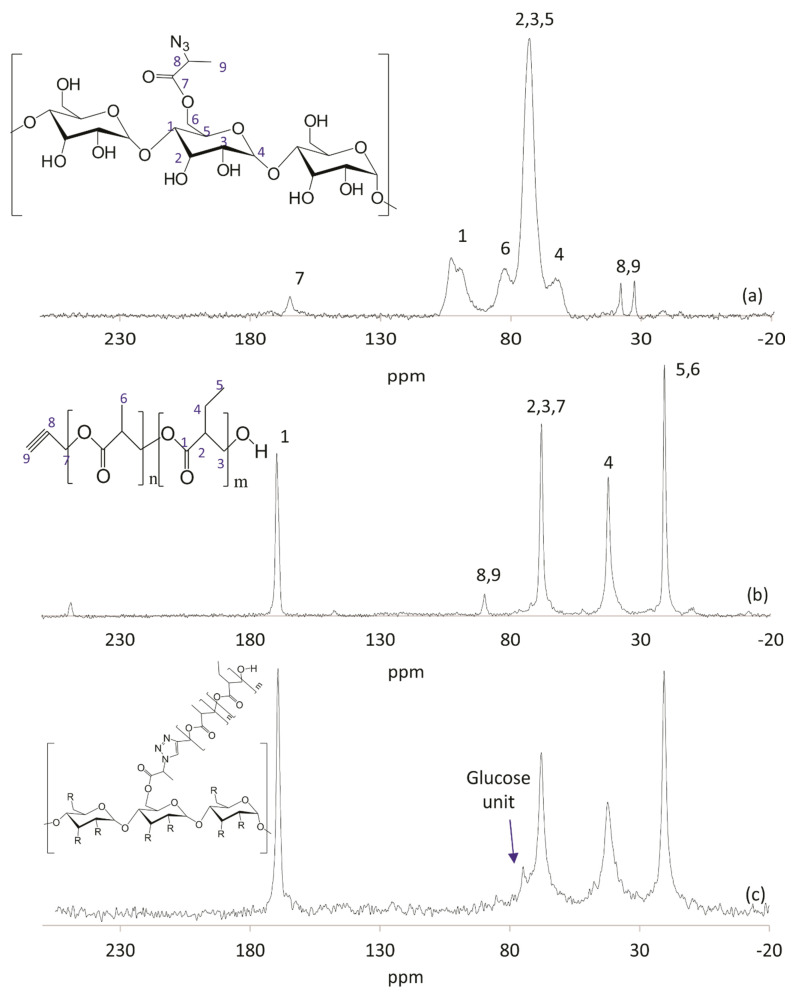
110,5 MHz solid-state ^13^C NMR spectra of PullN_3_ [29] (**a**), PHBHV-alkyne (**b**) and Pull-*g*-PHBHV (**c**).

^13^C NMR spectra of PullN_3_ (Figure 4a) and Pull-*g*-PHBHV (Figure 4c), both compared with the ^13^C NMR spectrum of PHBHV-alkyne (Figure 4b), showed resonances before and after the cycloaddition reaction. The resonances (ppm) at 101.1 and 98.5, from anomeric carbon in *α*(1→4)- and *α*(1→6)-linked glucose units of PullN_3_ (Figure 4a) are less evidenced in the Pull-*g*-PHBHV spectrum shown in Figure 4c, and this occurs mainly because of two reasons: the overlapping of PHBHV peaks in the same region and the greater noise presented by the grafted polymer analysis signal. The analysis had to be performed in solid-state, due to the insolubility of Pull-*g*-PHBHV in the deuterated solvents used for this technique. However, it can be noticed the presence of a predominant peak from PullN_3_ at 72.2 ppm in the copolymer spectrum (Figure 4c). Besides that, the resonance at 89.4 ppm from the alkyne carbon of the propargyl termination of PHBHV disappeared after its grafting onto pullulan, indicating the success of the click chemistry reaction between the azide and the alkyne.

**Figure 5:**
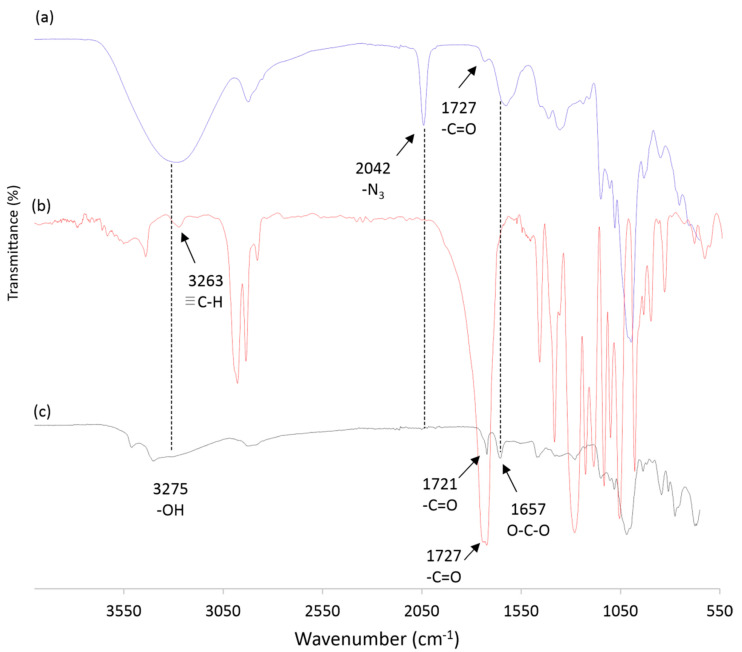
FTIR spectra of PullN_3_ (**a**), PHBHV-alkyne (**b**) and Pull-*g*-PHBHV (**c**).

Analyzing the FTIR spectra of the azide-functionalized pullulan PullN_3_ [29] (Figure 5a) and Pull-*g*-PHBHV (Figure 5c), both compared with the PHBHV-alkyne FTIR spectrum (Figure 5b), it can be noticed the disappearance of the characteristic stretching related to the C-N_3_ bond at 2042 cm^−1^, when comparing Figure 5c with Figure 5a. This fact evidences the grafting of the PHBHV copolymer onto pullulan chains via click chemistry. In addition, the appearance of stretching at 3375 cm^−1^ corresponding to an –OH bond, in Figure 5c, characterizes the presence of the remaining pullulan hydroxyl groups in the obtained grafted copolymer. Finally, the axial stretching at 1722 cm^−1^ (–C=O) observed in Figure 5c is typical of carbonyl groups from PHBHV chains, as corroborated by Figure 5b. These results evidence the success of the click chemistry reaction.

**Figure 6:**
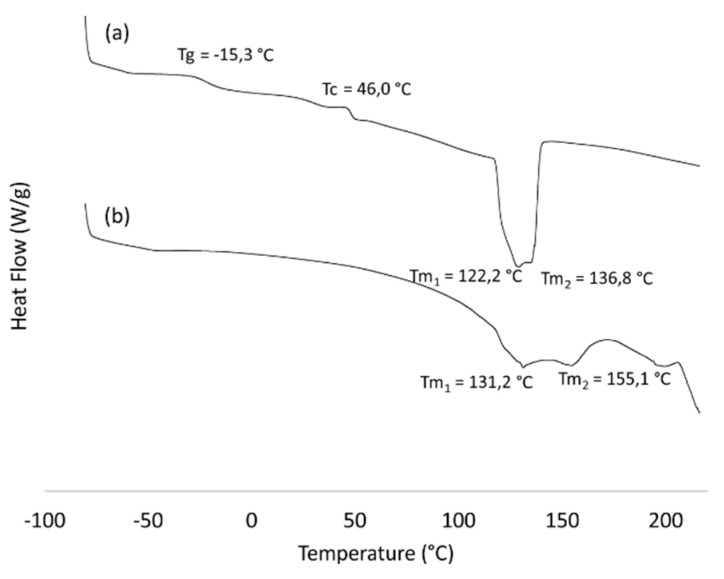
DSC thermograms of PHBHV-alkyne (**a**) and Pull-*g*-PHBHV (**b**).

**Figure 7:**
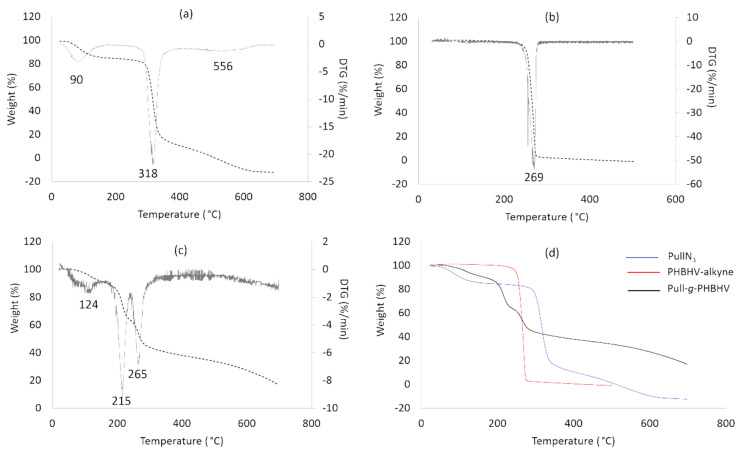
Thermogravimetric curves of PullN_3_ (**a**), PHBHV-alkyne (**b**) and Pull-*g*-PHBHV (**c**), with the respective derivatives, as well as correlation between the thermogravimetric curves of the functionalized polymers and the grafting product (**d**).

**Figure 8:**
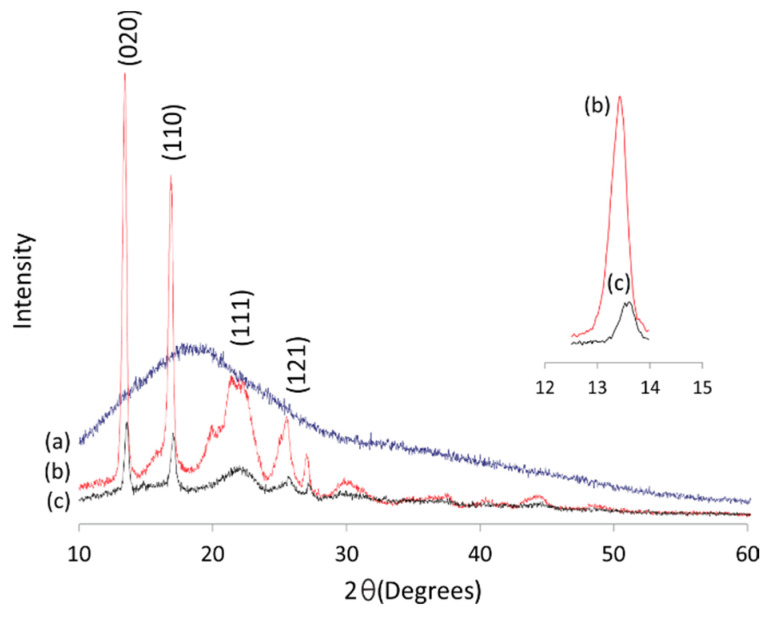
XRD diffractogram of PullN_3_ (**a**) PHBHV-alkyne (**b**) and grafted Pull-*g*-PHBHV copolymer (**c**).

Figure 6, Figure 7 and Figure 8, respectively, reveal the results concerning the thermal behavior (DSC), degradation (TGA) and crystallinity (DRX) of the prepared grafted copolymer Pull-*g*-PHBHV. DSC thermograms shown in Figure 6 reveal a different thermal behavior between the PHBHV-alkyne and grafted copolymer Pull-*g*-PHBHV. As previously discussed by Carvalho et al., 2020, PullN_3_ showed neither clear glass transition temperature (*Tg*) nor crystalline melting peak (*Tm*). This occurs due to the variability of pullulans’ properties which depends on the culture medium used for the production, and the tendency to retain humidity, especially for a hydrophilic polymer such as pullulan [29,34]. The PHBHV-alkyne thermogram revealed two melting peaks: at 122.2 and 136.8 °C, making the proximity of the values result in an overlapping of the peaks (Figure 6b). The presence of these two peaks is justified by the melting of crystals with different lamellar thicknesses or crystal structures, which occurs by a drastic temperature reduction resulting in the formation of crystals with low homogeneity [35]. The PHBHV melting–recrystallization–remelting, as well as the values found, are close to others previously reported in the literature [35,36]. When PHBHV chains were grafted onto pullulan, getting the Pull-*g*-PHBHV copolymer, a significant change in the melting point was observed. In addition, the decrease in the summation of the PHBHV-alkyne melting enthalpy peaks alongside the disappearance of the crystallinity peak (*Tc*) in Figure 6b when compared to the value of 46.0 °C in Figure 6a corroborate with the statement that PHBHV crystallinity was affected after its grafting onto pullulan amorphous chains.

Thermogravimetric analyses of the polymers used in the click chemistry reaction, and their reaction product, enabled an evaluation of the thermal stability of the materials. The pullulan behavior shown in Figure 7a presented three stages of mass loss: (i) the first when Tmax equals 90 °C is due to vaporization of the moisture absorbed by the hydrophilic material, (ii) the second peak when Tmax equals 318 °C is due to the disintegration of the pullulan macromolecular chains and, finally, (iii) the third when Tmax equals 556 °C results from the degradation of the polymer by-products, that is, the saccharide rings [18,37]. In Figure 7b, the PHBHV-alkyne behavior showed a single thermal decomposition stage, at 269 °C, which mainly occurs by a nonradical random chain scission mechanism involving a six-membered ring transition state [36]. For the thermal degradation of the Pull-*g*-PHBHV copolymer, three degradations were observed in three stages of mass loss, at 124, 215 and 265 °C, respectively. Comparing all the obtained thermograms, it is clear that Pull*-g-*PHBHV presents lower degradation temperature values, indicating its minor thermal stability. The presence of amine groups from the 1,2,3-triazol may enhance the copolymer degradation, as the decomposition products, amines or acidic protons could increase the random chain scission reaction of polyhydroxyalcanoate [38].

The XRD analysis confirmed the partial crystallinity of the PHBHV-alkyne, showing characteristic peaks of the material, such as (020), (110), (111) and (121) (Figure 8b) [39]. However, after its grafting onto pullulan chains, whose amorphous structure is shown in the diffractogram of Figure 8a, a reduction in the crystallinity peaks of the Pull-*g*-PHBHV grafted copolymers could be observed (Figure 8c), compared with the starting PHBHV-alkyne. The remaining less intense crystallinity peaks in Figure 8c characterize the presence of the PHBHV grafted Pull-*g*-PHBHV structure. This intensity reduction can be attributed to the presence of pullulan in the material.

## 4. Conclusions

In this work, the polysaccharide pullulan was chemically modified by grafting poly(3-hydroxybutyrate-*co*-3-hydroxyvalerate) onto its backbone via click chemistry. We used previously prepared azide-functionalized pullulan and synthesized PHBHV-alkyne via a transesterification reaction. The functionalized polymers were then conjugated via CuAAC for the synthesis of Pull-*g*-PHBHV. The chemical structure of the grafted copolymer was characterized by ^13^C NMR and FTIR techniques. Further analyses were conducted to demonstrate the modification in the thermal and crystallinity index of the materials, such as DSC, TGA and XRD techniques. Therefore, by the CuAAC reaction, a novel material has been developed from well-known bio-derived and biodegradable polymers, resulting in a final copolymer with interesting properties for further applications in biomedical applications, such as biodegradability and reduced crystallinity. The CuAAC methodology has the advantages of controlling both the amount and size of the polymer being grafted onto the pullulan’s backbone, varying according to the degree of substitution obtained from the polysaccharide modification. Besides that, this copolymer synthesis method guarantees the remaining of hydroxyl groups from pullulan without further modification, keeping the polysaccharide’s hydrophilic properties. Furthermore, the polymers used for the synthesis of this new graft copolymer, pullulan and PHBHV, are both biocompatible and biodegradable, making possible the development of a unique material with great potential for biomedical devices. Finally, it is important to highlight that the Pull-*g*-PHBHV grafted copolymer is an innovative biopolymer, carefully prepared considering its future applications in the elaboration of particles for the encapsulation of active ingredients and evaluation of controlled release systems. Therefore, Pull-*g*-PHBHV has good potential to be applied in the biomedical field, mainly as a drug delivery system.

## Figures and Tables

**Table 1:** Number average molar masses (*Mn*) and dispersity (*Đ*) of unmodified PHBHV and PHBHV-alkyne.

Sample	*M_n_*(Da) ^a^	*M_n_*(Da) ^b^	*Đ*
**Unmodified PHBHV**	-	114,873	2.86
**PHBHV-alkyne**	2843	4049	1.54

Determined by ^a 1^H NMR and ^b^ GPC.
